# Validity and reliability of the Tianyu climbing machine in maximal oxygen uptake testing

**DOI:** 10.3389/fphys.2026.1850118

**Published:** 2026-06-11

**Authors:** Fengkai Yang, Wenfeng Liu, Tianyu Wu, Hui He, Hongxing Xun

**Affiliations:** 1 Hunan Provincial Key Laboratory of Physical Fitness and Sports Rehabilitation, Hunan Normal University, Changsha, China; 2 Key Laboratory of Kinesiology Evaluation and Recovery of General Administration of Sport of China (Hunan Institute of Sports Science), Hunan Institute of Sports Science(Hunan Anti-Doping Agency), Changsha, Hunan, China; 3 Faculty of Health Sciences and Sports, Macao Polytechnic University, Macao, Macao SAR, China

**Keywords:** cardiorespiratory fitness, exercise physiology, maximum oxygen, reliability, validity

## Abstract

**Background and aims:**

accurate assessment of maximum oxygen uptake (VO_2_max) is fundamental to exercise physiology and cardiorespiratory fitness evaluation. While treadmills are the gold standard, there is a growing need for standardized vertical loading devices. This study aimed to evaluate the concurrent validity, construct validity, and reliability of the novel Tianyu climbing machine during progressive load exercise testing.

**Methods and results:**

Thirty-one male physical education students (mean age: 20.45 years) were recruited to perform maximal graded exercise tests on both a treadmill (using the Bruce protocol) and the Tianyu climbing machine (starting at 0.05 m/s with 0.01 m/s increments every minute). Physiological indicators, including VO_2_max, heart rate (HR), and respiratory exchange ratio (RER), were monitored. Concurrent validity was assessed using Bland-Altman plots and mean relative percentage error (MRPE), while construct validity was examined through canonical correlation analysis (CCA). Test-retest reliability of the machine’s speed was evaluated via intraclass correlation coefficients (ICC).The climbing machine demonstrated high concurrent validity with the treadmill, with a mean relative percentage error (MRPE) of less than 10%.CCA revealed a significant linear relationship between physiological markers and climbing performance indicators (*r* = 0.779, *P* < 0.01). Furthermore, the device exhibited excellent speed accuracy (error < 1%) and high test-retest reliability (ICC = 0.74 - 0.85) across standardized speed ranges.

**Conclusion:**

The Tianyu climbing machine is a scientifically valid and reliable tool for assessing cardiorespiratory function. Its ability to provide precise, controllable vertical loads makes it an effective alternative to traditional modalities for standardized metabolic testing in athletic and clinical settings.

## 1 Introduction

For modern people, climbing remains an important form of exercise and a way of life; for instance, climbing stairs can improve cardiovascular function (Allison et al., 2017),outdoor rock climbing allows people to immerse themselves in nature and challenge their physical limits (Baláš et al., 2014),and strong climbing abilities are essential for emergency evacuation (Halder et al., 2021). In-depth investigation of energy expenditure during human climbing, and the acquisition of valuable physiological parameters, can provide valuable insights for health promotion, the enhancement of athletic performance, and emergency evacuation. However, conducting research on climbing as a form of exercise presents numerous practical difficulties, such as requiring participants to wear portable cardiopulmonary monitors while repeatedly ascending and descending a given route (Kozma and Pontzer, 2021).Consequently, it is necessary to develop an effective and reliable device capable of simulating natural climbing patterns.

To address this practical challenge, engineers have developed a range of climbing devices to study energy expenditure, muscle activation sequences and movement patterns during climbing, such as indoor rotating climbing walls (Limonta et al., 2018)and artificial rock vertical climbing walls (Kozma and Pontzer, 2021).In terms of power measurement, some climbing machines use hydraulic controllers to adjust the load, with users passively adapting to the resistance by turning a knob or altering their pedaling frequency (Lee and Deitrick, 1994).However, such equipment largely relies on non-linear resistance systems, meaning the load cannot be precisely quantified. Furthermore, the absence of mandatory speed constraints results in subjects of different weights experiencing varying actual intensities under the same resistance settings. This makes it difficult to achieve the progressive load increases typical of standard treadmills, thereby hindering the stable induction of physiological limit responses. Consequently, the development of a climbing machine capable of providing a stable climbing speed remains a challenging task.

Based on the principle that muscles perform mechanical work by overcoming gravity during human climbing, we have designed a novel climbing machine (National Invention Patent: ZL202011385278.7). The core objective of this design is to achieve ‘forced speed standardization’ through a servo motor system. A constant speed not only serves as a prerequisite for accurately quantifying vertical power (P = mg × v) and calibrating metabolic cost, but also transforms random movement rhythms into a controllable experimental load, thereby providing the hardware foundation for equivalent comparison with the ‘gold standard’ treadmill. This study, which focused on healthy participants, utilized this novel climbing machine to conduct maximal oxygen uptake (VO_2_ max) testing. It primarily assessed the test’s concurrent validity and construct validity across various metrics, while also investigating the equipment’s speed accuracy and test-retest reliability at different speeds. The aim was to provide a scientific basis for the standardized use and multi-scenario application of this novel climbing machine.

## 2 Materials and methods

### 2.1 Participants

Thirty-one healthy male physical education students were recruited based on strict inclusion criteria. The mean ± standard deviation of the participants’ baseline characteristics are as follows: age 19.82 ± 0.78 years, height 175.53 ± 6.11 cm, weight 69.67 ± 8.38 kg, and the interval between the two tests was 7.5 ± 0.4 days.

### 2.2 Selection criteria

Participants were required to meet the following inclusion criteria: (1) aged between 18 and 21 years (i.e. approximately 19 years); (2) maintaining a consistent weekly lifestyle with regular sleep patterns, and no additional exercise load apart from training and classes organized by the university; (3) currently free from injuries, pain or neurological disorders affecting the upper or lower limbs; (4) no history of any significant sports-related injuries within the past six months that might affect their ability to complete the tests.

The basis for sample size selection in this study is as follows: (1) In three experiments (K), the expected upper limit of the intraclass correlation coefficient (ICC) is in the range of > 0.85 to 0.9, with an 80% probability of obtaining a confidence interval width of 0.2 (Borg et al., 2022). (2) When the sample size is five times the total number of indicator variables analyzed (Filho and Toebe, 2022), the estimation bias of the typical correlation coefficient in CCA can be kept within an acceptable range (Caliński et al., 2006). (3) Based on the age-related patterns of human climbing ability, this study selected the age corresponding to the peak of this ability (approximately 19 years) as the age of the study subjects (Kraft, 2014).

Criteria for determining exhaustion during a maximum oxygen uptake test (Edvardsen et al., 2014):(1)The respiratory exchange ratio (R) is greater than 1.10; (2) The heart rate reaches the predicted maximum heart rate ± 10%; (3) The subject is unable to continue; (4) The oxygen consumption curve reaches a plateau or begins to decline (Bruce et al., 1973).

All participants were required to refrain from resistance training or high-intensity physical activity for 24 hours prior to testing. Prior to data collection, all participants completed a health screening questionnaire and signed an informed consent form.

### 2.3 Test procedures

All preparatory activities for the tests were conducted using a power bike. Participants were required to complete a 5 minute warm up at a load of 50 W set on the machine to achieve a state of physical readiness prior to testing.

**Figure 1:**
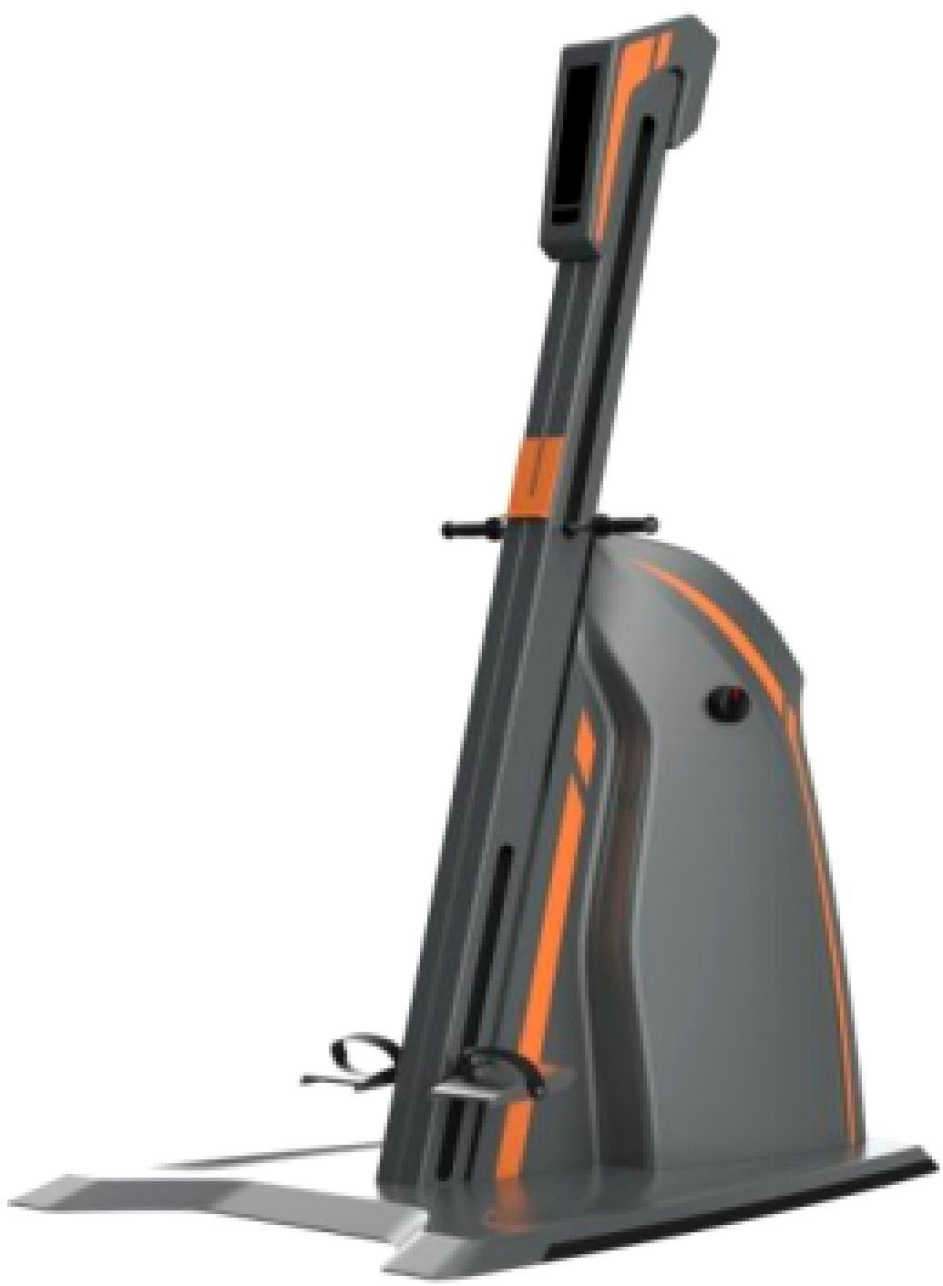
Tianyu climbing machine design drawing.

For validity testing, the same group of participants underwent maximum oxygen uptake tests on both a treadmill and the new climbing machine (as shown in **Figure 1**), with a one-week interval between the tests. All participants were randomly assigned to two groups: one group underwent the treadmill test followed by the climbing machine test, while the other group underwent the tests in the reverse order. Prior to the treadmill VO_2_max test, subjects were required to undergo a 3 minute acclimatization session on the treadmill (10% incline, 3 km/h speed, increasing by 1 km/h per minute). For the treadmill VO_2_max test, the exercise protocol adopted was as follows: the initial phase commenced at 2.7 km/h and 10% incline, followed by increments of 2% in incline at each stage, with speed increasing from 2.7 km/h to 4.0 km/h, 5.5 km/h, 6.8 km/h, 8.0 km/h, 8.9 km/h and 9.7 km/h, with a three-minute interval between each stage (Bruce et al., 1973).Prior to the VO_2_max test on the new climbing machine, subjects were required to perform a three-minute acclimatization session on the machine (at a speed of 0.05 m/s). The exercise protocol for VO_2_max measurement using the new climbing machine was as follows: an initial speed of 0.05 m/s, increasing by 0.05 m/s per minute (Kozma and Pontzer, 2021), with a three-minute interval between each stage. After each VO_2_max test, participants provided a self-reported rating of perceived exertion using the 20-point scale (Borg, 1982).

With regard to reliability testing, participants are required to undergo two identical tests using the climbing machine (with a one week interval between tests). During each test, participants must climb for a specified duration (30–60 seconds) at the speed provided by each gear setting on the climbing machine; upon completion, the experimenter records the time and distance covered. Participants must perform three tests at each speed setting. The interval between tests at each speed setting is one to two minutes (the duration is determined based on the participant’s condition). The criteria for determining exhaustion during the maximum oxygen uptake test follow the standard indicators established in classical research (Bruce et al., 1973; Edvardsen et al., 2014).

### 2.4 Experimental approach to the problem

This study demonstrates the concurrent validity of the Tianyu climbing machine by comparing the data obtained from it with that from a treadmill. It analyses the climbing parameters and physiological indicators generated during the measurement of maximum oxygen uptake using the Tianyu climbing machine to demonstrate its construct validity. Furthermore, by comparing the data from the two tests, the study determines the speed accuracy and test-retest reliability.

### 2.5 Data processing

To assess concurrent validity, the relative percentage error (%RE) and mean relative percentage error (MRPE) were calculated to determine the degree of deviation between the novel climbing machine and the gold standard (treadmill). Additionally, the root mean square error (RMSE) was employed to evaluate the consistency of the measurement results between the novel climbing machine and the gold standard (treadmill). Physiological parameters (such as VO_2_) were time-aligned to ensure consistency. Regarding speed accuracy, actual speed was derived by recording climbing distance and time, and compared with the target speed to calculate the Absolute Percentage Error (APE) and its mean (Mean Absolute Percentage Error, MAPE). Error classification criteria were set as poor (> 20%), moderate (10% – 20%), good (5% – 10%) and excellent (< 5%) (Read et al., 2021).

### 2.6 Statistical analysis

Concurrent validity was assessed through a visual analysis of the measurement results for the two devices using Bland-Altman plots, mean bias, and the limits of agreement for mixed effects (Parker et al., 2016).

Construct validity was assessed using a multi-stage correlation analysis: first, Pearson correlation analysis was used to examine preliminary relationships between variables; subsequently, partial correlation analysis was employed to observe the strength of the pure linear relationship between two variables after controlling for confounding variables; finally, canonical correlation analysis (CCA) was utilized to determine the overall linear relationship between the physiological indicators and the climbing performance indicators (Caliński et al., 2006).

A two-way random-effects model, absolute agreement, and intra-class coefficients (ICCs) based on multiple measurements (ICC_2_,K, where K represents the mean of three repeated measurements) were employed to assess the test-retest reliability of speed metrics on the novel climbing machine at approximately one-week intervals (Weir, 2005).Based on the upper and lower limits of the 95% confidence interval, the intraclass correlation coefficient values were classified as: poor (< 0.5), moderate (0.5 – 0.75), good (0.75 – 0.9) and excellent (> 0.9) (Koo and Li, 2016).he standard error of measurement (SEM), calculated as described in the data processing section, quantifies intra-individual measurement variability, while the minimum detectable change (MDC) is used to determine whether the change in a participant’s score between two tests exceeds the range of measurement error.

All raw data in this study were recorded simultaneously by a gas analyzer and the novel climbing robot, and were pre-processed, analyzed and visualized using RStudio 4.5.1 (Kousa Dogwood, Posit, Boston, USA).

## 3 Results

This study analyzed a total of 3 tests, 662 test sessions and 10 relevant parameters.

### 3.1 Concurrent validity

**Figure 2:**
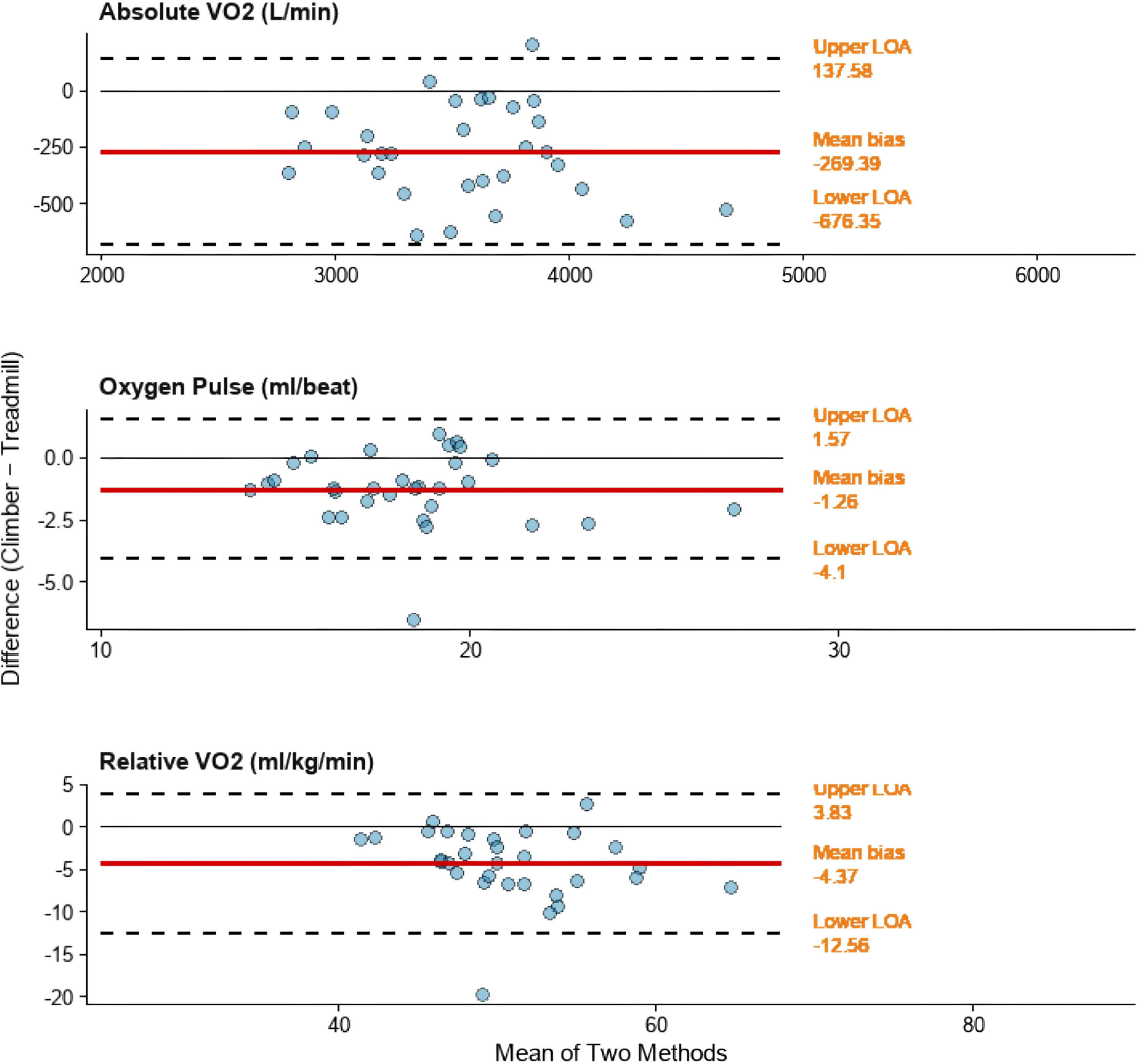
Concurrent validity BA plot.

The results of the gas metabolism analysis showed that the new climbing and treadmill systems demonstrated a high degree of consistency in terms of oxygen consumption, relative oxygen consumption and oxygen pulse (see **Table 1**). The mean relative percentage error (MRPE) for all physiological parameters was below 10%, falling within the acceptable range commonly used as a threshold standard in clinical decision-making. Bland-Altman analysis (**Figure 2**) further confirmed this consistency: the vast majority of data points were uniformly distributed within the 95% confidence limits, and the mean bias was stable, indicating that the device did not exhibit any significant systematic over- or underestimation.

**Table 1:** Concurrent validity.

Test	Metric	Concurrent validity		RMSE	MRPE	Interpretation
		Climbing machine Mean ± SD	Treadmill Mean ± SD			
VO2max Test	ml/min	3405.612903	3675	338.07	7.63%	Good
	ml/kg/min	48.62322581	52.98870968	6	8.27%	Good
	ml/HR	17.71419355	18.97903226	1.9	7.49%	Good

RMSE, root mean square error; MRPE, Mean Relative Percentage Error.

**Figure 3:**
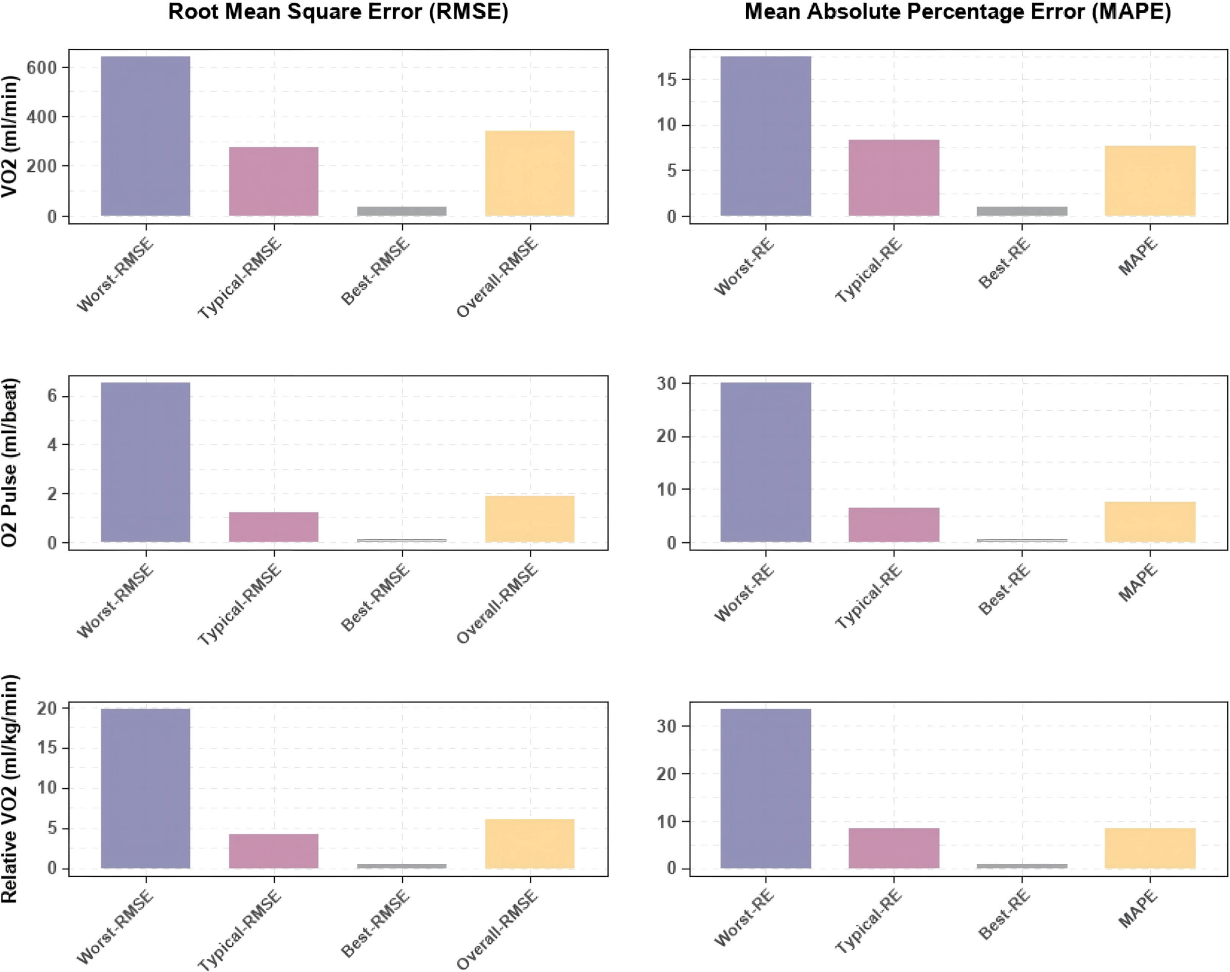
Bar chart showing the overall, worst, best, and typical values for each validity indicator.

Analysis of specific parameters (**Figure 3**) shows that the root mean square error (RMSE) for oxygen consumption was 338.07 ml/min (error of 7.63%), the RMSE for relative oxygen consumption was 6 ml/kg/min (error of 8.27%), and the root mean square error (RMSE) for the oxygen pulse was 1.9 ml/HR (error of 7.49%). The aforementioned error levels and stability indicators all confirm the accuracy of the device in capturing dynamic physiological responses during incremental exercise testing. The Borg scores obtained from climbing machine tests were consistently lower thanthose measured via treadmill testing (see **Table 2** for detailed data).

**Table 2:** Borg RPE score.

Assessment index	Climbing machine Mean ± SD	Treadmill Mean ± SD
General condition	16.72 ± 0.96	17.56 ± 0.75
cardiovascular condition	16.76 ± 0.95	17.24 ± 0.81
muscular condition	16.84 ± 1.12	17.8 ± 0.63

### 3.2 Construct validity

**Figure 4:**
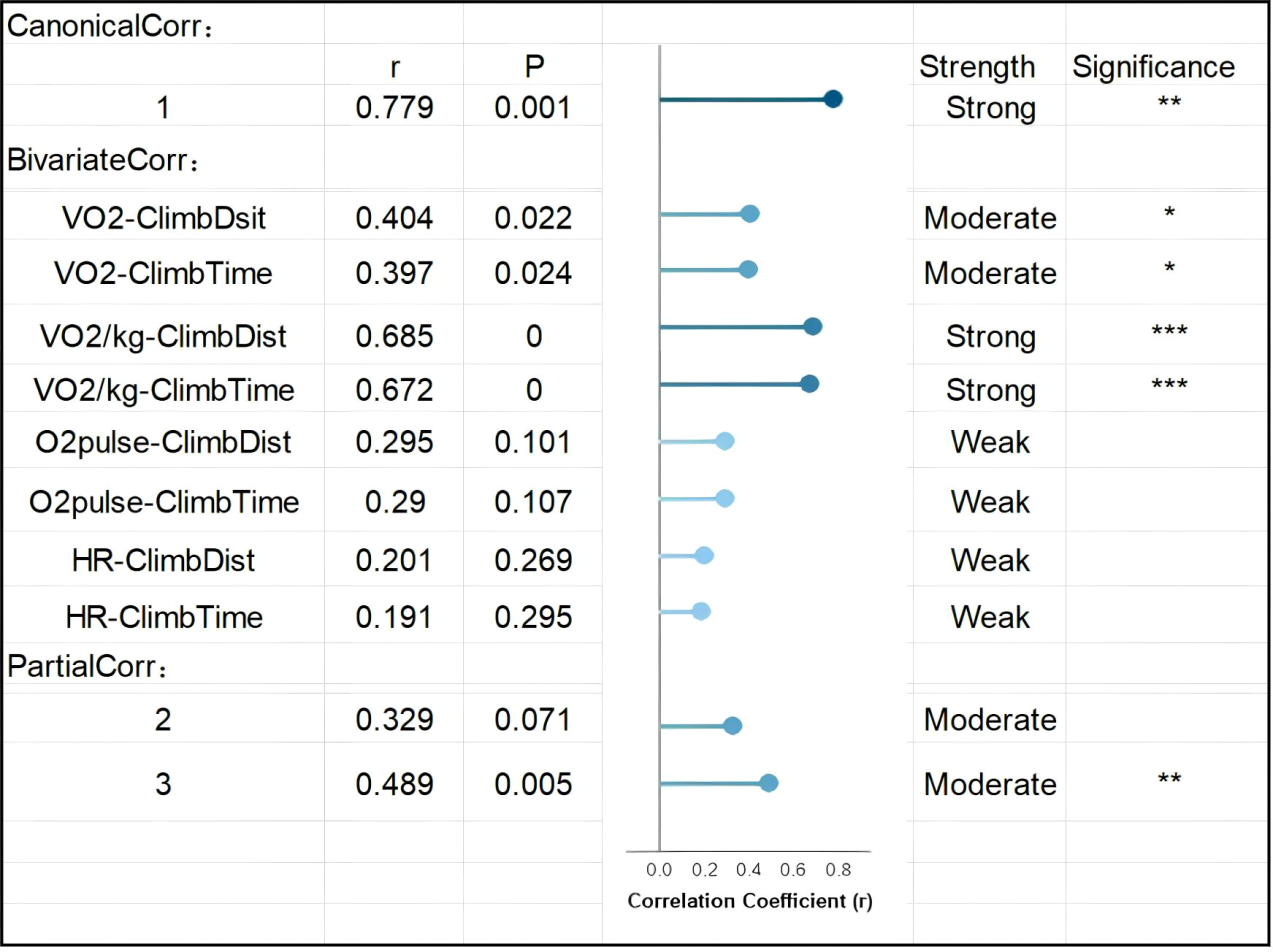
Construct validity: Forest plot of correlation coefficient. (*P<0.05, **P<0.01, ***P<0.001)1:First Canonical Correlation Coefficient 2:Climbing Time - Oxygen Pulse (Fixed Heart Rate) 3:Climbing Distance - Relative Oxygen Uptake (Fixed Climbing Time) Heart Rate:HR; Distance:Dist.

Canonical correlation analysis (CCA) revealed a significant and strong overall correlation between the climbing group’s indicators (climbing height and climbing time) and the physiological group’s indicators (oxygen consumption, relative oxygen consumption, oxygen pulse and heart rate) (first canonical correlation coefficient r = 0.779, P < 0.01; see **Figure 4**). In univariate analyses, relative oxygen consumption exhibited a significant and strong positive correlation with both climbing distance and time (r = 0.672 – 0.685, P < 0.001). Oxygen uptake showed a significant moderate correlation with climbing metrics (r ≈ 0.40, P < 0.05), while the direct correlation between oxygen pulse and heart rate did not reach statistical significance (P > 0.05). Partial correlation analysis further revealed the intrinsic relationships between the parameters: after controlling for heart rate, the oxygen pulse exhibited a significant positive correlation with climbing time (r = 0.489, P = 0.005).

### 3.3 Reliability

**Figure 5:**
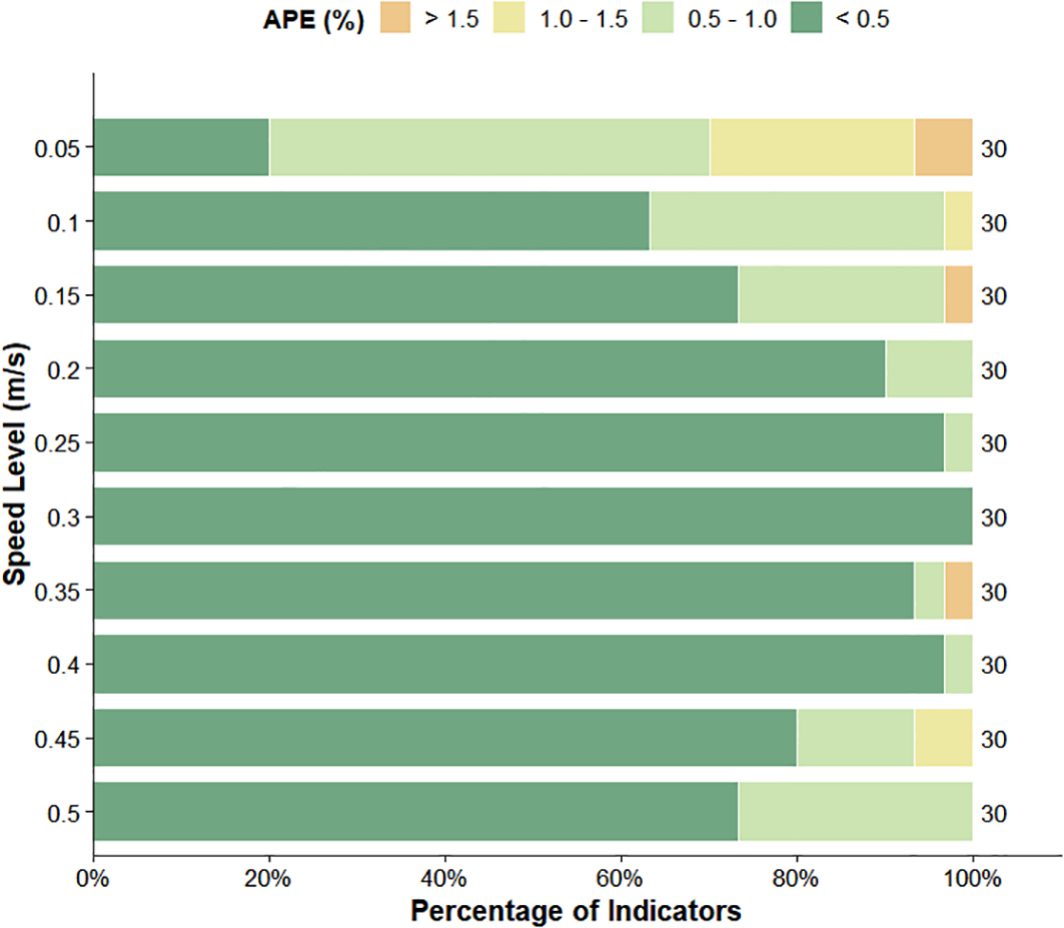
Reliability, percentage of an individuals absolute error relative to the total score across all tests.

**Figure 6:**
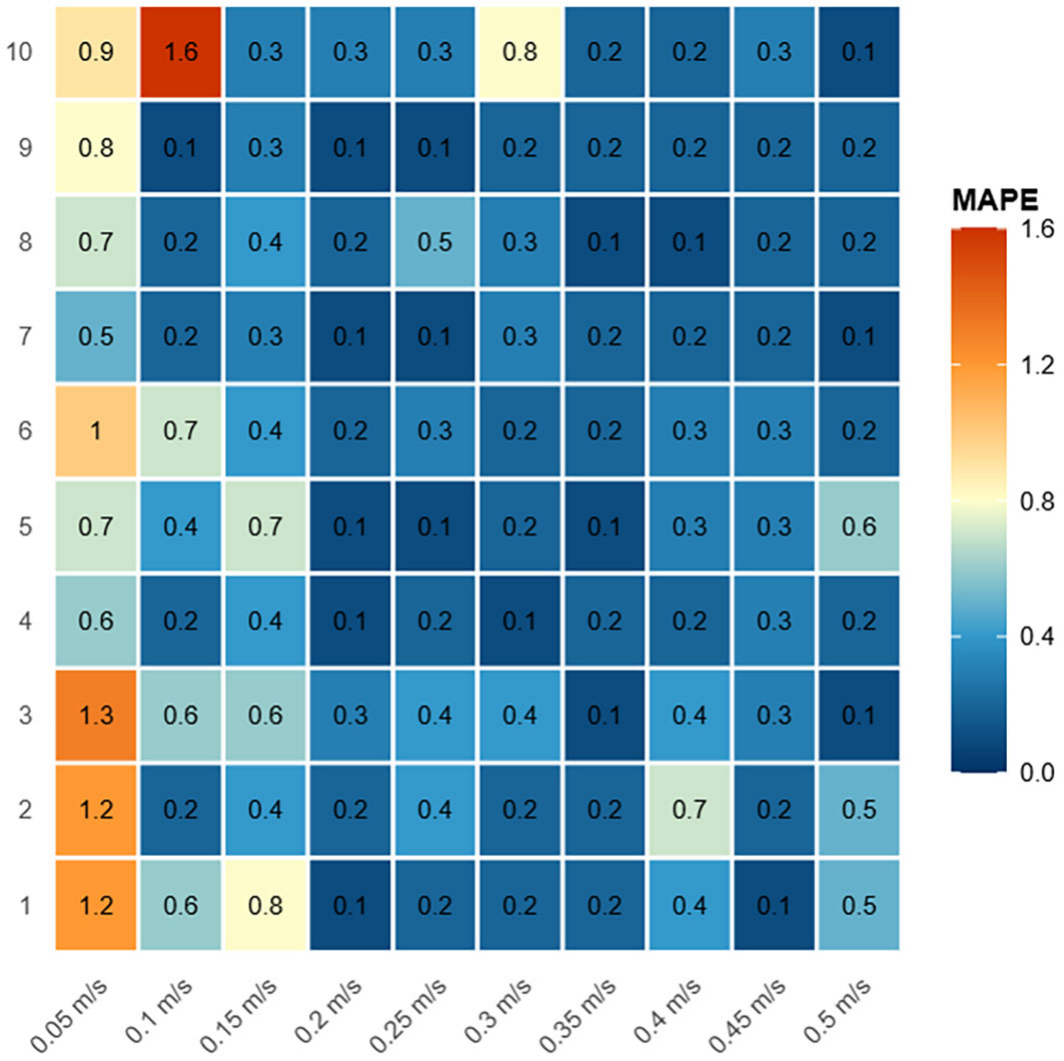
Reliability—Mean Absolute Percentage Error (MAPE)of the individuals three measurements.

**Figure 7:**
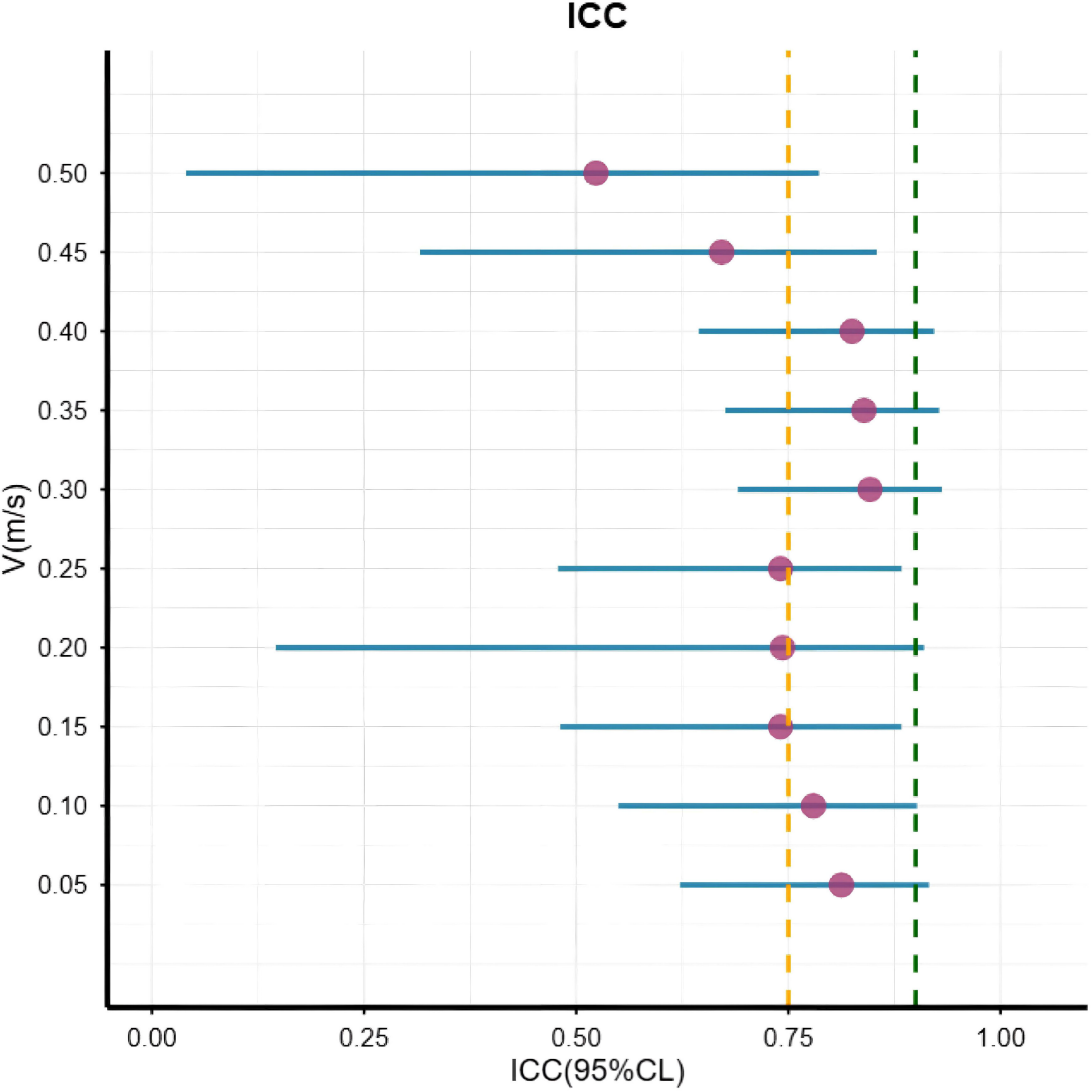
Reliability ICC(95%CL).

The new climbing machine demonstrated extremely high speed accuracy across all test speed ranges, with an absolute percentage error (APE) consistently below 5% (**Figure 5**), achieving an excellent standard, while the mean absolute percentage error (MAPE) remained below 1% (see **Table 3** and **Figure 6** for details). Data analysis (**Table 3**) indicates that retest reliability tests conducted at all speed ranges on the new climbing machine achieved moderate to good retest reliability. Within the commonly used speed range of 0.05 m/s to 0.4 m/s, test-retest reliability was stable and excellent. At very high speed settings (>0.45 m/s), the stability of the equipment showed a slight downward trend; although the average reliability remained within an acceptable range, the lower limit of the 95% confidence interval widened (**Figure 7**). Furthermore, the standard error of measurement (SEM) and minimum detectable change (MDC) for each speed range remained at low levels, confirming the stability of the device in repeated measurements.

**Table 3:** Test-retest reliability and speed-accuracy evaluation.

TEST	Metric	Test-retest reliability		ICC (95%CL)	SEM	MDC(95%CL)	Interpretation	Speed-accuracy evaluation	
		DAY1 Mean ± SD	DAY2 Mean ± SD					MAPE (allover)	MAPE (individual)
0.05 Vtest	m/s	0.0505 ± 0.0002	0.0503 ± 0.002	0.81 (0.62~0.92)	0.00014	0.00039	Moderate-Excellent	0.87	(0-2)
0.1 Vtest	m/s	0.1005 ± 0.006	0.1004 ± 0.002	0.78 (0.55~0.90)	0.00017	0.00047	Moderate-Good	0.47	(0.3-1.3)
0.15 Vtest	m/s	0.1501 ± 0.0022	0.1505 ± 0.009	0.74 (0.48~0.88)	0.00012	0.00034	Poor-Good	0.43	(0-3.8)
0.2 Vtest	m/s	0.2002 ± 0.008	0.1998 ± 0.005	0.74 (0.15~0.90)	0.00053	0.0015	Poor-Good	0.27	(0-0.9)
0.25 Vtest	m/s	0.2500 ± 0.0005	0.2513 ± 0.0055	0.74 (0.48~0.88)	0.00017	0.00048	Moderate-Good	0.16	(0-0.5)
0.3 Vtest	m/s	0.3000 ± 0.0007	0.2994 ± 0.0003 ±	0.85 (0.69~0.93)	0.00011	0.00031	Moderate-Excellent	0.18	(0-0.3)
0.35 Vtest	m/s	0.3498 ± 0.0010	0.3489 ± 0.0008	0.84 (0.68~0.93)	0.0002	0.00055	Moderate-Excellent	0.28	(0.1-0.7)
0.4 Vtest	m/s	0.3996 ± 0.0021	0.3989 ± 0.0004	0.83 (0.64~0.92)	0.00024	0.00068	Moderate-Excellent	0.24	(0.1-0.6)
0.45 Vtest	m/s	0.4495 ± 0.0014	0.4500 ± 0.0014	0.67 (0.32~0.85)	0.0012	0.0034	Poor-Good	0.29	(0-1.1)
0.5 Vtest	m/s	0.4995 ± 0.0011	0.5016±0.0016	0.52 (0.04~0.79)	0.0039	0.01	Poor-Good	0.27	(0-1)

1: Based on the mean of 3 trials per participant; SD, standard deviation; 95%CI, 95% confidence interval; ICC, intraclass coefficient; MDC, minimal detectable change; SEM, standard error of measurement;. MAPE, Mean Absolute Percentage Error.

## 4 Discussion

This study confirmed the validity and reliability of the new climbing machine in VO_2_max testing. Correlation analysis revealed a strong correlation (*r* = 0.779) and a low MRPE (< 10%), indicating that this device can serve as a valid alternative to a treadmill.

### 4.1 Concurrent validity

The results obtained in this study are consistent with those reported by Lee et al. (Lee and Deitrick, 1994)using the VersaClimber, both confirming that physiological parameters measured on the climbing machine are highly correlated with those obtained on a treadmill, making it an effective alternative tool for assessing human cardiorespiratory function. However, the VO_2_max results obtained in this study were consistently lower on the climbing machine than on the treadmill, whereas the findings of Lee et al. were the opposite. This inconsistency may stem from two factors: firstly, population-specific adaptation; the subjects in this study were sports science undergraduates who possessed a higher level of sport-specific neuromuscular adaptation to treadmill exercise, enabling them to delay local fatigue more effectively; secondly, issues regarding equipment accuracy: the new climbing machine utilizes servo motors to achieve more precise speed and resistance control, eliminating the additional compensatory work caused by resistance fluctuations in earlier mechanical devices, thereby providing more accurate data. Furthermore, the influence of body weight should not be overlooked; as vertical climbing is a typical weight-bearing exercise, the metabolic demand is highly sensitive to the subject’s mass. The relatively uniform weight profile of our participants may have minimized the variance in mechanical efficiency, though it also limits our understanding of the machine’s performance across a broader weight spectrum.

### 4.2 Construct validity

This study employed Canonical Correlation Analysis (CCA) to examine the relationship between physiological indicators and climbing indicators, revealing a significant overall linear association (r = 0.779, P = 0.001). This result indicates a strong multivariate association between the two sets of variables. Specifically, the incremental exercise loads prescribed by the climbing machine testing protocol elicit a corresponding cascade of internal physiological adaptations, which are captured by the machine’s output metrics with high systemic synchronicity. The strength of this canonical correlation demonstrates that the variations in the device’s recorded climbing metrics are strictly coupled with the subjects’ metabolic strain throughout the loading procedure.

Rather than implying that physiological indicators directly determine climbing outcomes, this finding reflects a consistent correspondence between internal physiological responses and externally measured climbing indicators. Within the climbing indicators, variables such as climbing distance—derived from the device-set speed over time—represent the external work performed. The observed associations therefore suggest that the outputs generated by the device correspond well with physiological responses at the level of statistical relationships, supporting its construct validity in reflecting exercise-related characteristics.

In terms of indicator specificity, relative oxygen consumption showed a stronger association with climbing indicators (r > 0.67) than absolute oxygen consumption and heart rate, suggesting its greater relevance in characterizing physiological responses during gravity-defying exercise. In contrast, the weaker associations observed for heart rate and oxygen pulse may be related to non-linear heart rate dynamics or baroreflex responses induced by isometric muscle contractions during climbing, which can attenuate their statistical relationship with oxygen consumption (Stien et al., 2022; Hanna et al., 2008).

Partial correlation analysis further showed that, after controlling for climbing time, the association between climbing distance and relative oxygen consumption was reduced, indicating that these variables share variance related to exercise duration. Additionally, after accounting for heart rate, the association between oxygen pulse and time increased (r = 0.489), suggesting that oxygen pulse may capture aspects of physiological response not fully reflected by heart rate alone. This implies that during the climb, the increase in heart rate is disproportionate to the increase in oxygen consumption; this phenomenon may be caused by a sympathetic-mediated pressor response triggered by metabolic reflexes (Giles et al., 2006).

Overall, these findings indicate that the climbing indicators generated by the device exhibit consistent statistical associations with physiological indicators. This alignment supports the potential utility of the climbing machine as a tool for assessing individual physical capacity, based on the observed relationships between external workload-related metrics and internal physiological responses.

### 4.3 Reliability

This study confirms that the new climbing machine achieves an excellent level of speed accuracy, with an overall average absolute error percentage of less than 1% and individual absolute error percentages kept within 5%, demonstrating its significant potential as a new evaluation tool. In terms of reliability, the device performed robustly in the 0.05 – 0.4 m/s range (ICC: 0.74 – 0.85); however, in the high-speed range above 0.45 m/s, the ICC values dropped to 0.67 – 0.52, indicating challenges to measurement stability. In response to this fluctuation, and based on a combination of literature and experimental observations, the following two core mechanisms warrant particular attention: Firstly, regarding the interference caused by extreme loads, this finding is similar to the results of the study by Hao-ran Qu et al (Qu, 2024), in which the Jueying device they developed experienced slight interference to its measurement stability under an 80% 1 RM load due to the extreme load, indicating that extreme loads pose a certain challenge to precision monitoring environments. Similarly, our climbing machine exhibits an upward trend in SEM and MDC 95% CL values as speed increases, implying that errors are greater during high-speed testing and that more pronounced changes are required to be recognized as genuine alterations. Secondly, regarding the issue of the critical point of human coordination, based on experimental observations, the sudden drop in reliability is not entirely due to mechanical precision; rather, it is largely because, at speeds exceeding this threshold, the human body may have reached the critical point of coordination for the climbing movement. At this point, the frequency of upper-body pulling and lower-body pedaling exceeds the subject’s habitual range of motion, leading to increased displacement of the center of mass (Sousa and Tavares, 2015).The above results not only confirm the challenge of maintaining stable output from the equipment under high-speed conditions, but also suggest that in future research or training applications, assessment speeds should be kept below the stability threshold (0.4 m/s) wherever possible, or that appropriate error compensation schemes should be developed for the high-speed range.

### 4.4 Limitations of the study

To evaluate the generalizability of the device, we recruited participants accustomed to land-based running. However, the observed VO_2_max results may have been influenced by several neuromuscular constraints. First, in terms of muscle involvement, climbing demands intense systemic coordination of both upper and lower limb groups, unlike the primarily lower-body focus of running (Giles et al., 2006). Second, the unique muscle recruitment patterns required for vertical climbing—including different joint kinematics and increased isometric components—can induce premature local fatigue in individuals who are not habituated to this modality (Halder et al., 2018). Third, due to the participants’ established exercise habits in running, a lack of specialized neuromuscular adaptation to the Tianyu machine likely caused them to reach peripheral exhaustion before attaining their true central cardiorespiratory aerobic limit. This discrepancy in adaptation may account for the lower VO_2_max values obtained with the device compared to treadmill measurements. Therefore, future research should incorporate professional climbers or athletes with specialized movement adaptations to more accurately evaluate the device’s physiological specificity and its applicability in elite performance contexts.

The sample consisted of only 31 young, physically active men, lacking representation of female participants, broader age groups, and clinical populations. This relatively small and homogeneous sample limits the generalizability of the findings. Future studies should include larger and more diverse cohorts to further evaluate the applicability of the Tianyu climbing machine across different populations.

In this study, measurement errors reached the centimeter or even millimeter level; when calculating the corresponding percentage errors, variables with smaller units may amplify these errors, potentially resulting in calculated values that exceed the actual error margins.

## 5 Conclusions

Through multidimensional validity testing and reliability analysis, the scientific validity and reliability of the new climbing machine in providing progressive resistance exercise and assessing human cardiopulmonary function have been demonstrated. The machine is capable of delivering precise progressive resistance, and its monitored indicators accurately reflect the user’s physiological function, making it an effective scientific tool for assessing human cardiopulmonary function and athletic performance.

## Data Availability

The original contributions presented in the study are included in the article/**Supplementary Material**. Further inquiries can be directed to the corresponding author.
