# Supplementary material for: Validity and reliability of the Tianyu climbing machine in maximal oxygen uptake testing
Source: Front Physiol. 2026 Jun 11;17:1850118. doi: 10.3389/fphys.2026.1850118 (PMC13295003; doi:10.3389/fphys.2026.1850118)
Supplement: Supplementary file 1 [file DataSheet1.docx]

(Equipment Description): The climbing machine used in this study is a novel device that has been granted a national invention patent (ZL202011385278.7). This Tianyu climbing machine simulates human climbing movements, enabling constant-speed vertical motion and accurately measuring climbing height; its core relies on a servo motor system to perform real-time monitoring of climbing distance and stable regulation of the target climbing speed.

**Schematic diagram of the novel climbing machine structure**

Working principle of the new climbing machine: This device connects the footplate and handle via left and right guide rods housed within guide columns. The guide rods are coupled to a one-way bearing sprocket on the main shaft via a chain. During the downward phase, the one-way bearing locks, and the motor-driven system regulates the downward speed of the guide rods; during the upward phase, the movement of the guide rods is not constrained by the main shaft. To maintain a constant vertical height, the user must overcome their own body weight to perform the upward climbing motion. A servo motor precisely monitors the climbing speed, thereby enabling real-time calculation of climbing power (power = force × speed). Furthermore, a counterweight system connected via pulleys offsets the weight of the moving components whilst also working in conjunction with a displacement sensor to monitor the user’s centre of gravity.


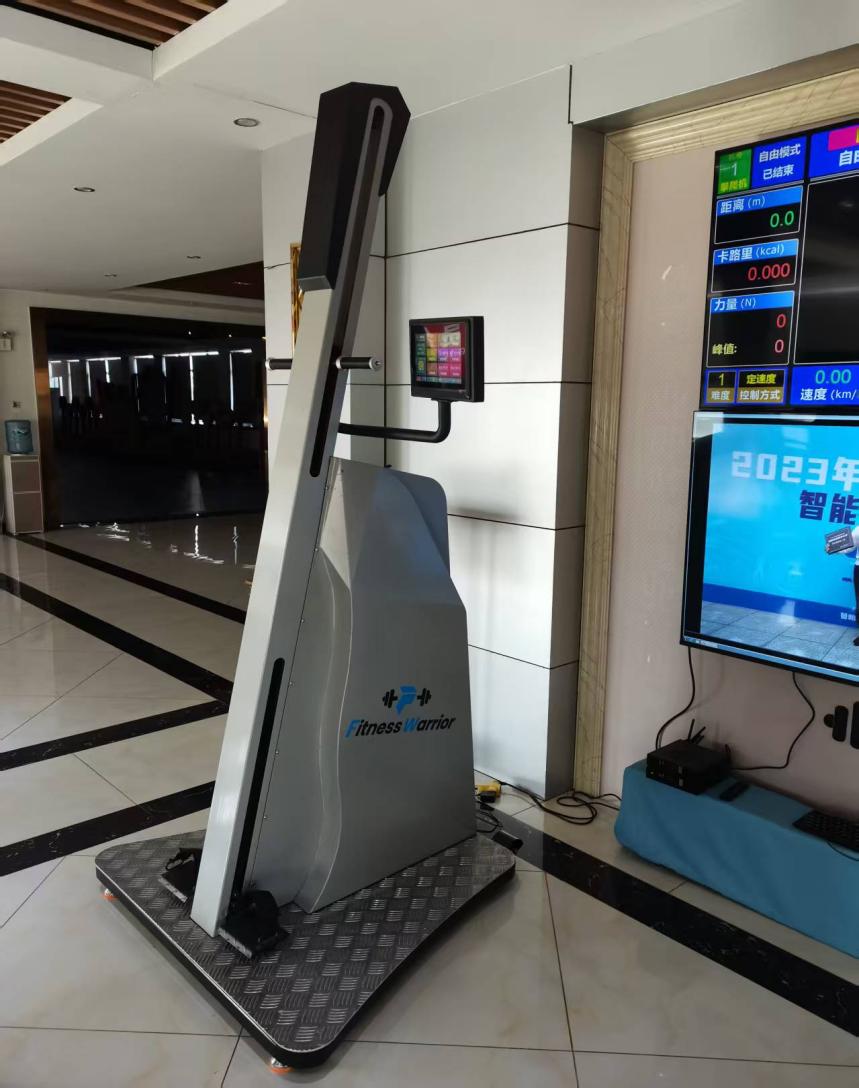


**External view of the climbing machine**

**Supplementary1. Means and standard deviations for each construct validity measure；APE values for each participant at each speed in terms of reliability**

| **Table supplementary 1.** |  |  |
| --- | --- | --- |
| **Construct Validity** |  |  |
|  | Metric | Mean ± SD |
| climbing height | m | 218.80 ± 32.86 |
| climbing time | s | 1161.06 ± 93.39 |
| oxygen consumption | ml/min | 3405.61 ± 416.74 |
| relative oxygen consumption | ml/kg/min | 48.62 ± 4.96 |
| oxygen pulse | ml/HR | 17.71 ± 2.63 |
| heart rate | bpm | 191.26 ± 8.82 |
| **Speed-Accuracy Evaluation** |  |  |
| **APE %** |  |  |
| **0.05 Vtest** | % |  |
| Participants |  |  |
| 1 |  | 1.2 |
| 2 |  | 1.2 |
| 3 |  | 1.3 |
| 4 |  | 0.6 |
| 5 |  | 0.7 |
| 6 |  | 1 |
| 7 |  | 0.5 |
| 8 |  | 0.7 |
| 9 |  | 0.8 |
| 10 |  | 0.9 |
| **0.1 Vtest** | % |  |
| Participants |  |  |
| 1 |  | 0.8 |
| 2 |  | 0.4 |
| 3 |  | 0.6 |
| 4 |  | 0.4 |
| 5 |  | 0.7 |
| 6 |  | 0.4 |
| 7 |  | 0.3 |
| 8 |  | 0.4 |
| 9 |  | 0.3 |
| 10 |  | 0.3 |
| **0.15 Vtest** | % |  |
| Participants |  |  |
| 1 |  | 0.6 |
| 2 |  | 0.2 |
| 3 |  | 0.6 |
| 4 |  | 0.2 |
| 5 |  | 0.4 |
| 6 |  | 0.7 |
| 7 |  | 0.2 |
| 8 |  | 0.2 |
| 9 |  | 0.1 |
| 10 |  | 1.6 |
| **0.2 Vtest** | % |  |
| Participants |  |  |
| 1 |  | 0.2 |
| 2 |  | 0.4 |
| 3 |  | 0.4 |
| 4 |  | 0.2 |
| 5 |  | 0.1 |
| 6 |  | 0.3 |
| 7 |  | 0.1 |
| 8 |  | 0.5 |
| 9 |  | 0.1 |
| 10 |  | 0.3 |
| **0.25 Vtest** | % |  |
| Participants |  |  |
| 1 |  | 0.1 |
| 2 |  | 0.2 |
| 3 |  | 0.3 |
| 4 |  | 0.1 |
| 5 |  | 0.1 |
| 6 |  | 0.2 |
| 7 |  | 0.1 |
| 8 |  | 0.2 |
| 9 |  | 0.1 |
| 10 |  | 0.3 |
| **0.3 Vtest** | % |  |
| Participants |  |  |
| 1 |  | 0.2 |
| 2 |  | 0.2 |
| 34 |  | 0.1 |
| 5 |  | 0.2 |
| 6 |  | 0.1 |
| 7 |  | 0.2 |
| 8 |  | 0.2 |
| 9 |  | 0.1 |
| 10 |  | 0.2 |
| **0.35 Vtest** | % | 0.2 |
| Participants |  |  |
| 1 |  | 0.2 |
| 2 |  | 0.2 |
| 3 |  | 0.4 |
| 4 |  | 0.1 |
| 5 |  | 0.2 |
| 6 |  | 0.2 |
| 7 |  | 0.3 |
| 8 |  | 0.3 |
| 9 |  | 0.2 |
| 10 |  | 0.8 |
| **0.4 Vtest** | % |  |
| Participants |  |  |
| 1 |  | 0.1 |
| 2 |  | 0.2 |
| 3 |  | 0.3 |
| 4 |  | 0.3 |
| 5 |  | 0.3 |
| 6 |  | 0.3 |
| 7 |  | 0.2 |
| 8 |  | 0.2 |
| 9 |  | 0.2 |
| 10 |  | 0.3 |
| **0.45 Vtest** | % |  |
| Participants |  |  |
| 1 |  | 0.4 |
| 2 |  | 0.7 |
| 3 |  | 0.4 |
| 4 |  | 0.2 |
| 5 |  | 0.3 |
| 6 |  | 0.3 |
| 7 |  | 0.2 |
| 8 |  | 0.1 |
| 9 |  | 0.2 |
| 10 |  | 0.2 |
| **0.5 Vtest** | % |  |
| Participants |  |  |
| 1 |  | 0.5 |
| 2 |  | 0.5 |
| 3 |  | 0.1 |
| 4 |  | 0.2 |
| 5 |  | 0.6 |
| 6 |  | 0.2 |
| 7 |  | 0.1 |
| 8 |  | 0.2 |
| 9 |  | 0.2 |
| 10 |  | 0.1 |
